# Muscular involvement in Sjögren’s disease is associated with male gender, Ro52 autoantibodies, and a higher clinical activity

**DOI:** 10.3389/fmed.2026.1720681

**Published:** 2026-05-06

**Authors:** Christine Gründges, Sabrina Benz, Anna Meinecke, Liane Gaedecke, Antoine Murray, Tabea Seeliger, Franz Felix Konen, Thomas Skripuletz, Torsten Witte, Nadine Zehrfeld, Diana Ernst

**Affiliations:** 1 Department of Rheumatology and Clinical Immunology, Hannover Medical School, Hannover, Germany; 2 Department of Psychology, Ludwig-Maximilians-Universität München, Munich, Germany; 3 Department of Neurology and Clinical Neurophysiology, Hannover Medical School, Hannover, Germany

**Keywords:** inflammatory myositis, muscular involvement, myopathy, Sjögren’s disease, Sjögren’s syndrome, SSA/Ro52-antibodies

## Abstract

**Background:**

Sjögren’s disease (SjD) is a systemic autoimmune disease that primarily affects exocrine glands. It can be associated with a variety of systemic manifestations, including myositis. At present, data evaluating muscular inflammation in SjD is limited, and further investigation is required.

**Methods:**

Patients with SjD and European Alliance of Associations for Rheumatology (EULAR) Sjögren’s syndrome disease activity index (ESSDAI) muscular involvement (EMI) of at least six points in the muscular domain were recruited retrospectively from Hannover Medical School’s SjD cohort after muscular inflammation was ascertained. Patients with SjD and EMI were characterised and compared with age- and sex-matched controls and with the entire SjD cohort.

**Results:**

A total of 26 of 492 SjD patients (5%) were diagnosed with EMI. The mean age of EMI onset was 56.4 years, the ratio between males and females was 12:14. Compared to SjD patients without EMI, SjD patients with EMI showed higher frequencies of Sjögren’s Syndrome-related Antigen A/Ro(52) [SSA/Ro(52)] antibodies (*p* < 0.001) and elevated levels of immunoglobulin G (*p* = 0.022). Additionally, ESSDAI scores at initial diagnosis (*p* < 0.001) and during follow-up (*p* = 0.003), and the number of immunosuppressive drugs taken over the course of treatment (*p* < 0.001), were significantly higher in SjD patients with EMI compared to those without muscular inflammation, as shown in the matched analysis, corroborated by the unmatched comparison.

**Conclusion:**

EMI is a rare but severe manifestation of SjD, occurring simultaneously with sicca symptoms. Male sex, positive SSA/Ro(52) antibodies, and high disease activity are more common in SjD patients with inflammatory myositis compared to those without muscular involvement.

## Introduction

Sjögren’s disease (SjD) is a systemic autoimmune disorder, originating from lymphocytic inflammation of exocrine glands (1). Its prevalence is reported to range between 1:100 and 1:400 in the literature, with a marked female predominance, particularly around the climacteric period (2). SjD primarily involves lacrimal and salivary gland dysfunction, leading to sicca symptoms (3, 4). However, extraglandular involvement is also common in SjD and may result in systemic disease manifestation (5, 6). For instance, cardiovascular, pulmonary, renal, gastrointestinal, and neurological involvement is known with regard to SjD (5–9).

European Alliance of Associations for Rheumatology (EULAR) Sjögren’s syndrome disease activity index (ESSDAI), muscular involvement (EMI) represents a rare and understudied extraglandular manifestation of SjD, occurring in approximately 1:100 SjD patients with a variety of muscular phenotypes (10–12). Thus, the current literature lacks a clear conceptual and nomenclatural distinction between primary SjD with myositis, myositis with associated SjD, and overlapping syndromes, complicating the understanding of muscular disease manifestations. Nevertheless, the majority of investigations have been conducted in presumed primary SjD (10–15).

In SjD, EMI typically presents with proximal muscle weakness and is often accompanied by myalgia (10). Felten et al. (11) demonstrated that antinuclear antibodies, rheumatoid factor, and increased levels of creatine kinase (CK) are frequently observed in patients with SjD and muscular involvement. Similar to myositis without SjD, electromyographic studies commonly reveal features indicative of muscular degeneration and inflammation, for example, spontaneous pathological activity and polyphasic motor unit potentials with reduced amplitude (10).

From a histopathological perspective, the majority of the cases of EMI in patients with SjD can be classified within the spectrum of inflammatory myopathies. The majority fall into either overlap myositis, historically often labelled as polymyositis, accounting for approximately 20–83% of cases, or inclusion body myositis (IBM), reported in 17–50% with varying rates due to small sample sizes and differences in study populations (10, 11, 15). Conventional immunosuppressive therapies, including glucocorticoids and methotrexate, appear to be effective in treating myositis associated with SjD (10–12). However, IBM seems to be an exception, as this myositis subtype does not respond well to immunosuppressive therapy, necessitating intensified myositis therapy (13). Arising from the high frequency of insufficient therapeutic response, current investigations are evaluating the significance of antibodies against cytosolic 5′-nucleotidase 1A in IBM and their possible association with myositis diagnosis in SjD (12).

With regard to the characteristics of SjD in patients with EMI, Colafransesco et al. (10) were able to demonstrate that SjD symptoms may present simultaneously with muscular manifestations. Analogous to SjD without EMI, typical disease manifestations include the inflammation of glandular structures, ocular and oral sicca symptoms, and the presence of Sjögren’s syndrome-related antigen A antibodies (alt. Robert antigen, SSA/Ro antibodies) in approximately 70% of the myositis patients examined (10). Compared to SjD patients without myositis, those with concomitant muscular inflammation tend to develop sicca symptoms approximately 10 years earlier, indicating a younger subgroup affected by SjD symptoms (11). Moreover, a markedly greater impairment in the quality of life was observed among patients with concurrent SjD and muscular inflammation (14). Against SjD patients without EMI, SjD patients with muscular manifestation are treated with 3-fold higher numbers of immunosuppressive agents, possibly reflecting a more systemic disease phenotype within this subgroup (11). There seem to be no apparent differences between SjD patients with and without EMI regarding sex distribution, extraglandular manifestation, and serological markers (rheumatoid factor, antinuclear antibodies, SSA/Ro antibodies, SSB/La antibodies), as shown in previous studies (11). Thus, younger age at disease onset remains the only distinguishing clinical indicator currently suggestive of coexisting EMI.

In patients with SjD, EMI constitutes a systemic manifestation that remains insufficiently characterised. The objective of our study was to delineate the clinical features and the optimal treatment of EMI in the context of SjD. Additionally, we compared SjD patients with and without EMI to identify potential indicators associated with muscular inflammation.

## Methods

### Study design and patient selection

**Figure 1:**
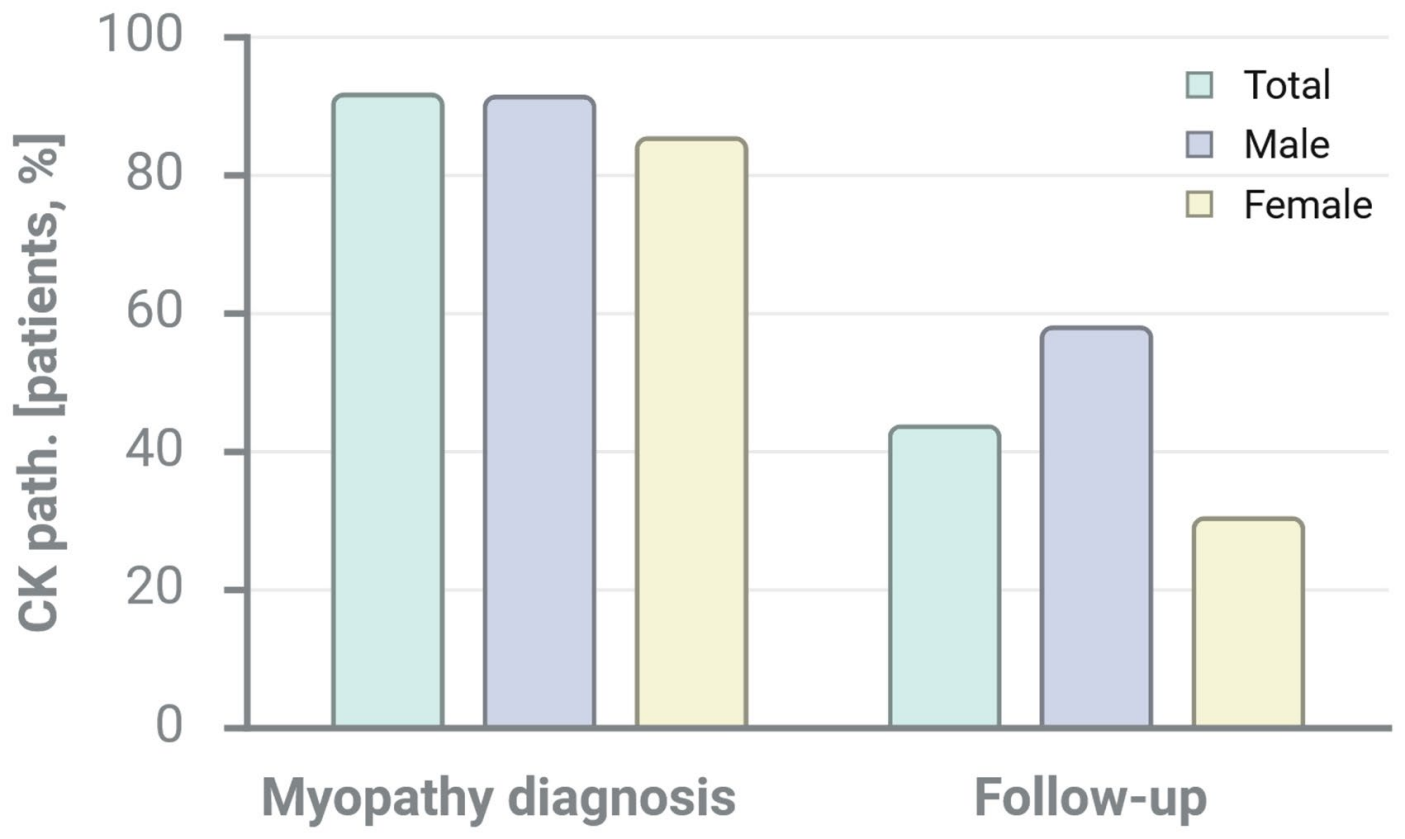
Study design. SjD: Sjögren’s disease, ESSDAI: EULAR Sjögren’s syndrome disease activity index; Gründges C, Zehrfeld N, created in BioRender 13 August 2025, licensed under BioRender publication license (https://biorender.com/r3zguxk).

This study was designed as a retrospective data analysis and is based on information collected from SjD patients treated at Hannover Medical School between 2015 and 2025 (c.f. Figure 1). SjD Patients were included when the American College of Rheumatology/ European Alliance of Associations for Rheumatology classification criteria of 2016 were fulfilled (16) and when a primary SjD was presumed. Given the exceptional rarity of EMI, five patients were included in the study, with SjD diagnoses established through expert clinical judgment, predominantly based on other typical SjD features like serological markers (antinuclear antibodies, SSA/Ro antibodies, SSB/La antibodies, alpha-Fodrin antibodies, hypergammaglobulinemia, lymphocytopenia) and clinical characteristics (sicca symptoms, suspicious salivary gland ultrasound).

**Figure 2:**
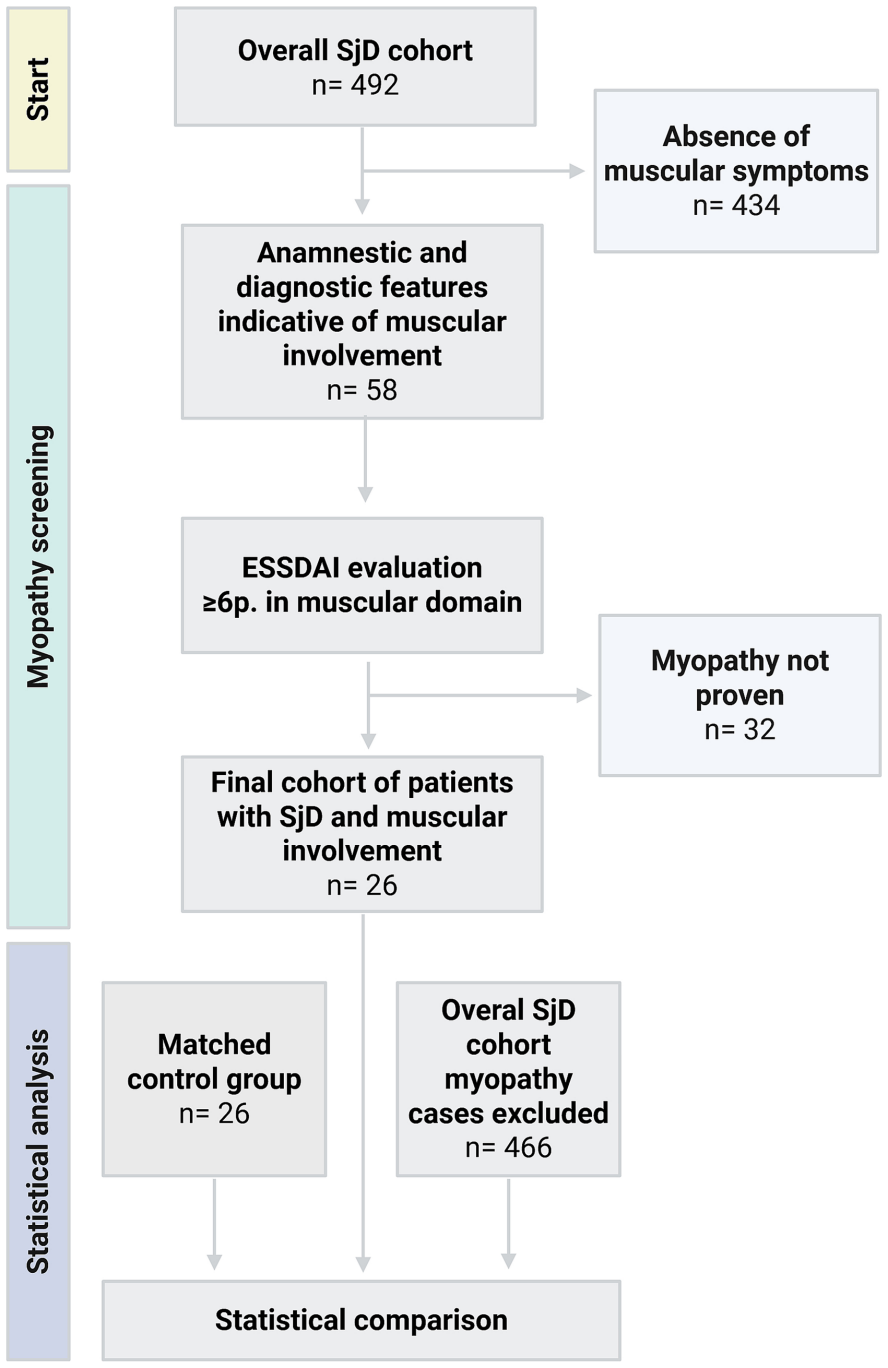
Muscular ESSDAI assessment. ESSDAI: EULAR Sjögren’s Syndrome Disease Activity Index (17), EMG: electromyography, MRI: magnetic resonance imaging, CK: creatine kinase; Gründges C, Zehrfeld N, created in BioRender 13 August 2025, licensed under BioRender publication license (https://biorender.com/flh4xi1).

To distinguish between true inflammatory myositis and non-specific muscle pain, EMI was assessed by applying the ESSDAI score (EULAR Sjögren’s syndrome disease activity index), which measures muscular disease activity on the basis of objective clinical, laboratory, and instrumental evidence (17). Accordingly, SjD patients were asked to score at least six points in the muscular ESSDAI domain, correlating with signs of myositis in electromyography, magnetic resonance imaging (MRI), or muscle biopsy. Based on the muscular ESSDAI score, isolated elevations of CK levels were considered an independent indicator of myositis only when exceeding twice the upper limit of normal, contributing to a score of 12 or 18 points (c.f. Figure 2).

Patients diagnosed with concomitant rheumatic diseases other than SjD (e.g., forms of associated SjD with overlapping features of SjD and rheumatoid arthritis, spondylarthritis, systemic lupus erythematosus, or systemic scleroderma) or patients with clinical signs of neoplastic SjD genesis were excluded from our investigations to avoid confounding variables within the SjD-EMI cohort. Additionally, patients with myopathy unrelated to inflammatory EMI (e.g., steroid or statin-induced myopathy, motor neuropathy) were excluded from our SjD-EMI subgroup.

### Data collection

Clinical data were obtained from Hannover Medical School’s SjD database as well as the university’s hospital information system. SjD patients diagnosed with associated EMI were characterised with regard to epidemiology, symptoms, laboratory parameters, diagnostic features, and therapeutic issues. To compare SjD patients with and without EMI, ESSPRI (EULAR Sjögren’s syndrome patient-reported index) and ESSDAI scores were reported, and SjD-specific diagnostic and therapeutic features were evaluated. Response to SjD therapy was assumed, if SjD patients achieved either MClI (minimal clinically important improvement = ESSDAI improvement ≥3 points) or PASS (patient acceptable symptom state = ESSPRI <5 points) (18). For EMI, response to therapy was defined as CK normalisation or a CK reduction of at least 50% and evaluated after individually scheduled follow-up.

### Statistical and graphical analysis

Statistical data analysis was performed in RStudio version 4.3.1, using SjD controls matched for age and sex and the university’s overall SjD cohort for comparison. All controls were diagnosed with primary SjD. Matching was conducted at a ratio of 1:1, with a median age difference of 1 year considered acceptable. Matched controls were selected randomly, therefore representing a cross-section of the hospital’s overall SjD cohort and being part of both subgroups. For statistical analysis, categorical variables were reported as absolute and relative frequencies and compared using Pearson’s chi-squared test or Fisher’s exact test, as appropriate. Continuous variables were summarised as means and standard deviations. Between-group comparisons were conducted using two-sample *t*-tests when assumptions of normality and homogeneity of variances (tested by Shapiro–Wilk and Levene’s tests) were met; otherwise, the non-parametric Wilcoxon rank-sum test was applied. Employing a two-tailed significance level of 5%, *p* < 0.05 was considered statistically significant. For graphic illustration, BioRender was used.

## Results

### EMI presentation in patients with SjD

EMI was diagnosed in 26/492 (5%) SjD patients. The mean age of SjD onset was 56.4 years, the average diagnostic delay between SjD and muscular manifestation was 0.3 years. Approximately half of the SjD patients diagnosed with inflammatory myopathy were male (46%).

In patients with SjD, common EMI symptoms included myalgia (76%) as well as subjective and bilateral muscle weakness (76 and 96%). EMI mainly presented as proximally emphasised tetraparesis (60%) or paraparesis of the lower limbs (24%) and was often accompanied by functional signs of muscular weakness (64%). Therefore, dysphagia (48%), dysarthria (8%), dyspnoea (24%), and myocarditis (16%) were recorded in SjD patients diagnosed with EMI.

The majority of SjD patients diagnosed with EMI presented with elevated CK levels (92%) and positive myositis antibodies (89%), particularly SSA/Ro52 antibodies (81%), at the time of inflammatory myopathy diagnosis. Additionally, 42% of the EMI cohort showed other myositis-specific antibodies, predominantly associated with dermatomyositis (MDA-5, SAE-2, NPX-2, and Mi-2a/ß), antisynthetase syndrome (Jo-1 and PL-7), and immune-mediated necrotising myopathy (signal recognition particle [SRP]). Approximately 35% of the myositis patients were characterised by positive SSA/Ro52 antibodies solely.

Among instrumental diagnostics, electromyography and magnetic resonance imaging were available in 19/26 and 18/26, respectively, patients and showed features indicative of EMI (spontaneous pathological activity and polyphasic motor unit potentials with reduced amplitude [electromyography], muscular oedema [magnetic resonance imaging]) in 74 and 89%, respectively. Muscle biopsy was performed in 15/26 SjD patients, with features of inflammatory myopathy in 87%. Aside from non-specific signs of inflammation and neurological changes, myopathies historically classified as polymyositis (40%) and IBM (13%) were the most common types of myositis in biopsy. While so-called polymyositis was typically indicated by endomysial inflammatory infiltrates, predominantly composed of CD8 + T-cells invading non-necrotic muscle fibres, IBM showed endomysial inflammation combined with degenerative features such as rimmed vacuoles and protein aggregates. in addition to myositis-specific diagnostics, nerve conduction studies were performed in 14/26 patients, with signs of concomitant polyneuropathy in 79%.

During the course of myositis treatment, SjD patients with muscular inflammation received an average of three additional immunosuppressive medications, glucocorticoids used for initial and bolus therapy excluded. Azathioprine, methotrexate, rituximab, and intravenous immunoglobulins (IVIGs) were administered most frequently. Importantly, these immunosuppressants were often used in combination for myositis therapy and selected individually based on therapeutic response and medication tolerability. 25/26 SjD patients with EMI received an individually scheduled follow-up for CK-evaluation with a mean time of assessment after 29.5 months. Measured by either CK normalisation or CK reduction of at least 50%, around 76% of SjD patients with myositis responded to therapy. Despite immunosuppressive treatment, 24% of SjD patients with muscular inflammation experienced stagnating or increasing CK levels; one patient could not be evaluated due to a lack CK values.

**Figure 3:**
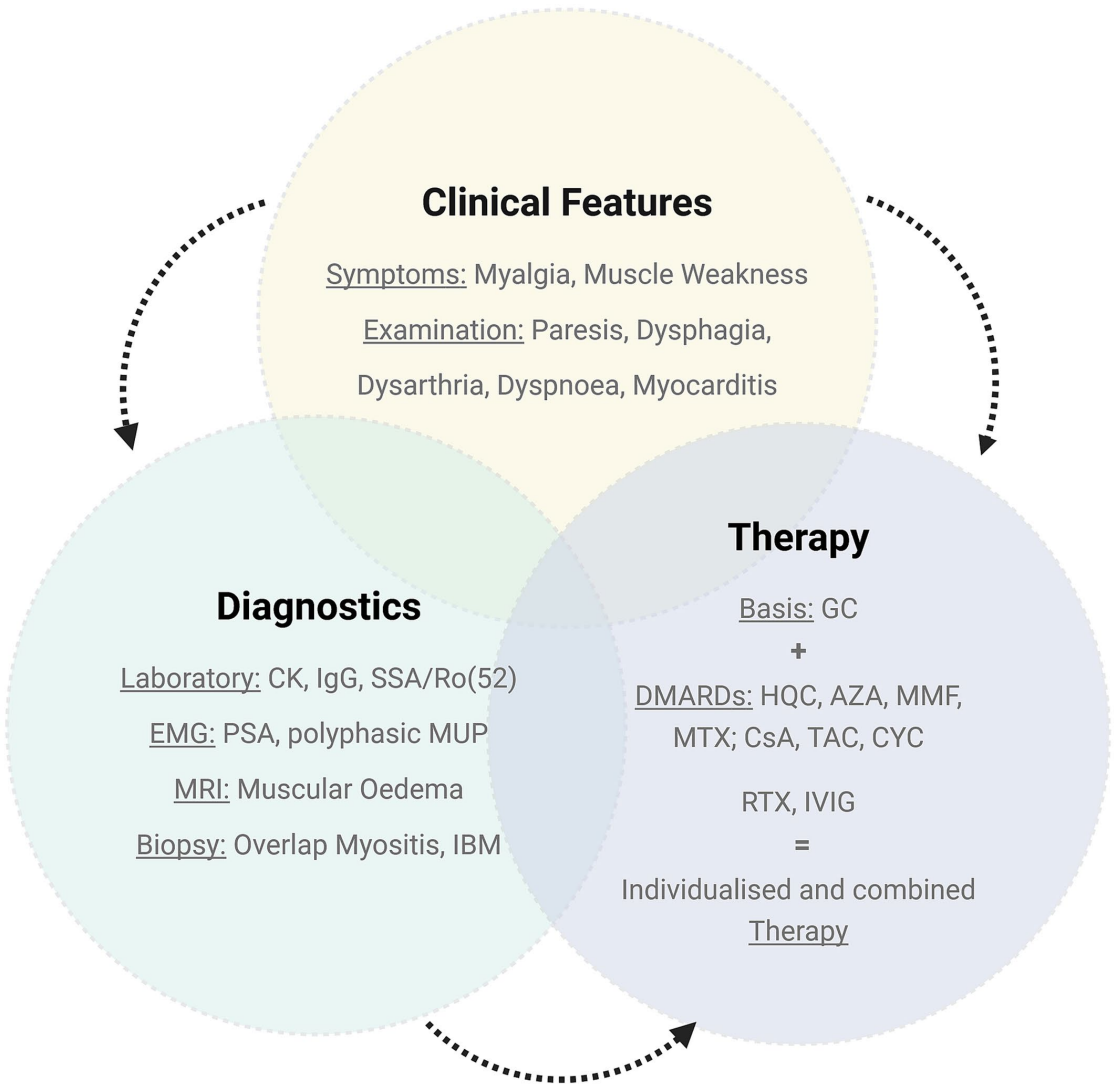
Myopathy characteristics in patients with SjD. CK: creatine kinase, EMG: electromyography, PSA: pathological spontaneous activity, MUP: motor unit potentials, MRI: magnetic resonance imaging, IBM: inclusion-body myosis, GC: glucocorticoids, DMARDs: disease modifying drugs, HCQ: hydroxychloroquine, AZA: azathioprine, MTX: methotrexate, MMF: mycophenolate mofetil, CsA: ciclosporin A, TAC: tacrolimus, RTX: rituximab, CYC: cyclophosphamide, IVIG: intravenous immunoglobulins; Gründges C, Zehrfeld N, created in BioRender 13 August 2025, licensed under BioRender publication license (https://biorender.com/hyhn0xy).

**Figure 4:**
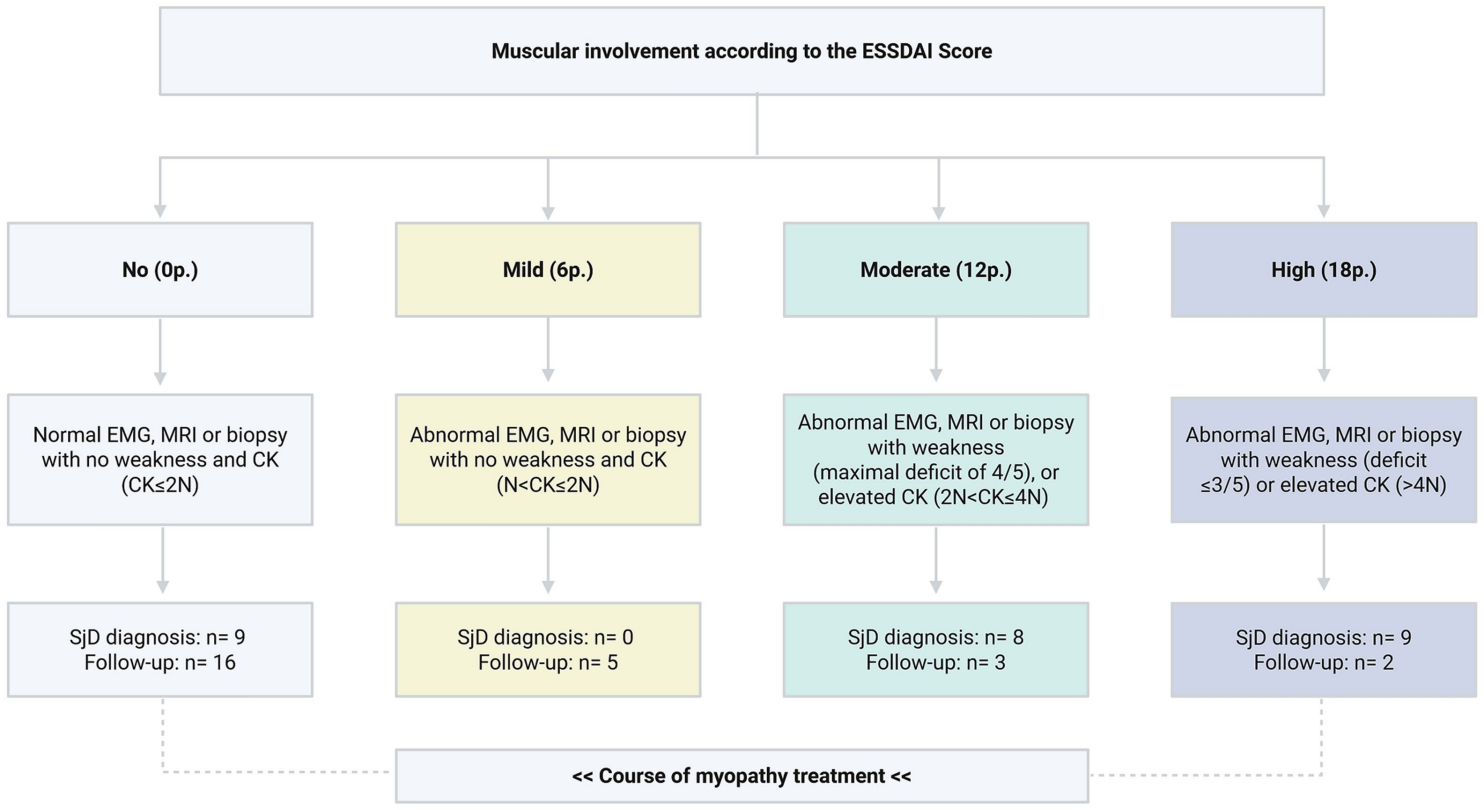
CK development during myopathy therapy. CK: creatine kinase; *CK values were evaluated for 25/26 patients and considered as pathological when measuring >170/145 U/L (m/f); Gründges C, Zehrfeld N, created in BioRender 13 August 2025, licensed under BioRender publication license (https://biorender.com/6x449m3).

Further information regarding the characterisation of myositis in SjD patients is shown in Table 1 and Figures 2–4. A more detailed description of SjD patients who are non-responders to myositis therapy is presented in Table 2.

**Table 1:** Characterisation of EMI in SjD (*n* = 26).

Epidemiology	
Age at SjD diagnosis [a], mean [SD]	56.4 (12.9)
Diagnostic delay SjD-EMI [a], mean [SD]	0.3 (3.1)
Sex ratio male/female [%]	12/14 (46/54)
Clinical presentation at EMI diagnosis and follow-up	
Myalgia, *n* [%]	19/25 (76)
Subjective muscle weakness, *n* [%]	19/25 (76)
Bilateral muscle weakness, *n* [%]	24/25 (96)
Proximally emphasised tetraparesis, *n* [%]	15/25 (60)
Proximally emphasised paraparesis of the lower limbs, *n* [%]	6/25 (24)
Functional signs of muscular involvement, *n* [%]	16/25 (64)
Dysphagia, *n* [%]	12/25 (48)
Dysarthria, *n* [%]	2/25 (8)
Dyspnoea, *n* [%]	6/25 (24)
Myocarditis, *n* [%]	4/25 (16)
Laboratory parameters at EMI diagnosis	
Leucocytes >10.2 tsd./μl, *n* [%]	6/25 (24)
C-Reactive protein >5 mg/L, *n* [%]	5/25 (20)
Creatine kinase >170/145 U/l (m/f), *n* [%]	23/25 (92)
Myositis antibodies path., *n* [%]	23/26 (89)
Myositis-specific antibodies, *n* [%]	11/26 (42)
SSA/Ro52 antibodies >6 U/mL, *n* [%]	21/26 (81)
Isolated positivity for SSA/Ro52 antibodies	9/26 (35)
Instrumental diagnostics at EMI diagnosis	
Electromyography pathological activity, *n* [%]	14/19 (74)
Magnetic resonance imaging pathological activity, *n* [%]	16/18 (89)
Muscle biopsy pathological activity, *n* [%]	13/15 (87)
Polymyositis/overlap myositis, *n* [%]	6/15 (40)
Inclusion-body myositis	2/15 (13)
Unspecific signs of inflammation, *n* [%]	4/15 (27)
Neurological changes, *n* [%]	5/15 (33)
Nerve conduction studies pathological activities, *n* [%]	11/14 (79)
Therapy ever during follow-up	
Hydroxychloroquine, *n* [%]	4/26 (15)
Azathioprine, *n* [%]	16/26 (62)
Methotrexate, *n* [%]	12/26 (46)
Mycophenolate mofetil, *n* [%]	9/26 (35)
Cyclosporin A, tacrolimus, *n* [%]	4/26 (15)
Rituximab, *n* [%]	11/26 (42)
Cyclophosphamide, *n* [%]	2/26 (8)
Intravenous immunoglobulins, *n* [%]	14/26 (54)
Response to therapy (normalisation of CK or reduction by at least 50%), *n* [%]	19/25 (76)

SjD, Sjögren’s disease; EMI, ESSDAI muscular involvement; bilat., bilateral.

**Table 2:** Case report of EMI patients without response to therapy.

Parameter	Patient 1	Patient 2	Patient 3	Patient 4	Patient 5	Patient 6
Epidemiology						
Sex	Female	Female	Male	Male	Female	Male
Age at SjD diagnosis	55	56	49	48	64	50
Age at EMI diagnosis	56	49	48	51	64	50
Diagnostic delay SjD-EMI	+	−	−	+	+/−	+/−
SjD characteristics						
ESSPRI at follow-up	7.3	6.0	1.0	6.7	8.3	0.0
ESSDAI at follow-up	5	14	18	30	31	16
Organ involvement other than muscle	−	−	−	+	+	+
Organ involvement				CNS	Lung	Lung PNS
EMI diagnostics						
Creatine kinase at EMI diagnosis (U/l)	402	307	1,291	911	260	280
Creatine kinase at follow-up (U/l)	679	311	765	643	324	286
SSA/Ro52 antibodies >6 U/ml	+	−	+	−	+	+
Electromyography path	+	−	−	+	+	
Magnetic resonance imaging path	+	+	−	+	+	
Muscle biopsy path	+	+		+	−	
Pattern in muscle biopsy	PM	PM		Not specified		
Nerve conduction studies path	+	−	−	+	+	−
Therapy						
Number of treatments (without GC)	4	5	1	4	3	3
Medication	HQC AZA MMF IVIG	HDQ MTX AZA MMF IVIG	AZA	MTX AZA RTX IVIG	HQC MTX AZA	MMF TAC RTX

SjD, Sjögren’s disease; EMI, ESSDAI muscular involvement; ESSPRI, EULAR Sjögren’s syndrome patient reported index; ESSDAI, EULAR Sjögren’s syndrome disease activity index; CNS, central nervous system; PNS, peripheral nervous system; PM, polymyositis; GC, glucocorticoids; HCQ, hydroxychloroquine; AZA, azathioprine; MTX, methotrexate; MMF, mycophenolate mofetil; TAC, tacrolimus; RTX, rituximab; IVIG, intravenous immunoglobulins.

### Comparison of SjD patients with and without EMI

In comparison to sex and age-matched SjD controls (c.f. Table 3), patients diagnosed with concomitant SjD and EMI were characterised by significantly higher ESSDAI scores at initial diagnosis (*p* < 0.001) and during follow-up (*p* = 0.003), but achieved MClI more often (*p =* 0.018). In addition, SjD patients with EMI showed higher detection rates of SSA/Ro(52) antibodies (*p* < 0.001) and SSB/La antibodies (*p* = 0.017) as well as elevated levels of immunoglobulin G (IgG, *p* = 0.022), likewise reflected in the biological ESSDAI score (*p* = 0.001). Moreover, SjD patients with muscular inflammation were treated with a markedly higher number of immunosuppressive drugs (*p <* 0.001). We could not detect differences in SjD patients with and without EMI regarding ESSPRI scores, organ involvement (ESSDAI domains other than the muscular and biological domain), and specific SjD diagnostics (salivary gland biopsy (available in 9/26 and 278/466 SjD patients), Saxon test, Schirmer’s test), when matching for sex and age.

**Table 3:** Comparison of SjD in patients with and without EMI.

Parameter	SjD with EMI (*n* = 26)	SjD without EMI, matched (*n* = 26)	(1)	Overall SjD cohort, unmatched (*n* = 466)	(2)
Epidemiology					
Age at SjD diagnosis [a], mean [SD]	56.4 (12.9)	56.3 (13.4)	n.s.	54.7 (15.4)	n.s.
Sex ratio male/female, [%]	12/14 (46/54)	12/14 (46/54)	n.s.	103/363 (22/78)	***p =* 0.010**
Muscular symptoms at follow-up					
Myalgia, *n* [%]	19/25 (76)	13/26 (50)	n.s.	195/440 (44)	***p =* 0.004**
ESSPRI at follow-up					
Dryness, mean [SD]	4.2 (2.7)	4.6 (3.3)	n.s.	4.7 (3.0)	n.s.
Fatigue, mean [SD]	5.3 (2.9)	4.8 (3.1)	n.s.	5.4 (3.2)	n.s.
Pain, mean [SD]	4.0 (3.6)	4.0 (2.9)	n.s.	4.8 (3.3)	n.s.
Score, mean [SD]	4.5 (2.5)	4.3 (2.5)	n.s	5.0 (2.5)	n.s.
ESSDAI at SjD diagnosis and follow-up					
Constitutional ≥ 2 patients, *n* [%]	9/26 (35)	3/26 (12)	n.s.	67/462 (15)	***p =* 0.013**
Lymphatic ≥ 4 patients, *n* [%]	2/26 (8)	2/26 (8)	n.s.	56/462 (12)	n.s.
Glandular ≥ 2 patients, *n* [%]	0/26 (0)	0/26 (0)	n.s.	28/462 (6)	n.s.
Articular ≥ 2 patients, *n* [%]	4/26 (15)	3/26 (12)	n.s.	100/462 (22)	n.s.
Cutaneous ≥3 patients, *n* [%]	0/26 (0)	1/26 (4)	n.s.	43/462 (9)	n.s.
Pulmonary ≥ 5 patients, *n* [%]	7/26 (27)	7/26 (27)	n.s.	64/462 (14)	n.s.
Renal ≥ 5 patients, *n* [%]	1/26 (4)	0/26 (0)	n.s.	15/462 (3)	n.s.
Muscular* ≥ 6 patients, *n* [%]	17/26 (65)	0/26 (0)	***p <* 0.001**	0/462 (0)	***p <* 0.001**
PNS ≥ 5 patients, *n* [%]	3/26 (12)	5/26 (19)	n.s.	182/462 (39)	***p* = 0.003**
CNS ≥ 10 patients, *n* [%]	1/26 (4)	1/26 (4)	n.s.	56/462 (12)	n.s.
Haematological ≥ 2 patients, *n* [%]	10/26 (39)	5/26 (19)	n.s.	97/462 (21)	n.s.
Biological ≥ 1 patient, *n* [%]	15/26 (58)	3/26 (12)	***p =* 0.001**	111/462 (24)	***p* < 0.001**
Score SjD diagnosis, mean [SD]	19.3 (13.0)	7.0 (7.2)	***p <* 0.001**	-	-
Score follow-up, mean [SD]	13.8 (9.1)	6.9 (7.7)	***p =* 0.003**	9.7 (8.1)	***p =* 0.014**
Diagnostics at SjD diagnosis					
Salivary gland biopsy FS ≥ 1, *n* [%]**	8/9 (89)	20/23 (87)	n.s.	264/278 (95)	n.s.
Saxon test ≤3.5 g/2 min, *n* [%]	12/22 (55)	13/26 (50)	n.s.	239/432 (55)	n.s.
Schirmer’s test ≤ 5 mm/5 min, *n* [%]	17/23 (74)	23/26 (89)	n.s.	352/449 (78)	n.s.
SSA/Ro antibody > 6 U/ml, *n* [%] SSA/Ro52 antibody > 6 U/ml, *n* [%] SSA/Ro60 antibody, *n* [%]	21/26 (81) 21/26 (81) 8/14 (57)	10/26 (39) 6/25 (24) 7/24 (29)	***p <* 0.001** ***p <* 0.001** n.s.	284/463 (61) - -	n.s. - -
SSB/La antibody > 6 U/mL, *n* [%]	10/25 (40)	2/26 (8)	***p =* 0.017**	99/457 (22)	n.s.
SSA/Ro + SSB/La AB path., *n* [%]	10/25 (40)	2/26 (8)	***p =* 0.017**	99/280 (35)	n.s.
IgG, Mean [SD]	16.3 (6.9)	13.4 (6.5)	***p =* 0.022**	13.9 (6.6)	***p =* 0.033**
Therapy during follow-up					
Number of drugs (without GC), mean [SD]	3.0 (1.4)	1.5 (1.3)	***p <* 0.001**	–	–
Response to therapy (PASS or MClI), *n* [%]	19/26 (73)	15/26 (58)	n.s.	–	–
PASS, *n* [%]	9/21 (43)	13/24 (54)	n.s.	–	–
MClI, *n* [%]	13/26 (50)	4/26 (15)	***p =* 0.018**	–	–

SjD, Sjögren’s disease; EMI, ESSDAI muscular involvement; ESSPRI, EULAR Sjögren’s syndrome patient reported index, ESSDAI; EULAR Sjögren’s syndrome disease activity index; PNS, peripheral nervous system; CNS, central nervous system; FS, Focus score according to Chisholm and Mason; GC, glucocorticoids; MClI, minimal clinically important improvement; PASS, patient acceptable symptom state; n.s., not specified; **Nine patients were asked to score ESSDAI muscular domain ≥6 in the course of SjD, *Salivary gland biopsy: available in 287/492 patients; (1,2): statistical significance proven in comparison with matched control group (1) or overall SjD cohort (2), bold values indicate statistical significance.

To avoid confounding from matching, we also compared patients diagnosed with SjD and EMI with the centre’s overall SjD cohort. In SjD patients with EMI, a markedly higher proportion of male patients (*p* = 0.010) was observed. Moreover, the unmatched comparison revealed higher scores for constitutional (*p* = 0.013), immunological (biological ESSDAI domain, *p* < 0.001; IgG *p* = 0.033), and overall (ESSDAI score at follow-up, *p* = 0.014) disease activity.

Supplemental data on additional differences are shown in Table 3.

## Discussion

### EMI presentation in SjD

We assessed patients with SjD and concomitant EMI, particularly focussing on epidemiology, clinical manifestation, diagnostics, therapeutic approaches, and patient outcomes. Overall, our findings correlate well with previous studies conducted in this context. More importantly, we identified new characteristics of muscular inflammation in patients with SjD, thereby facilitating myositis diagnosis.

Generally, our study shows that EMI remains a rather rare but severe manifestation of SjD, occurring in approximately 5:100 SjD patients. Consequently, we detected an EMI rate higher than reported in literature (10, 11, 13), possibly explained by the university’s broad service area and clinical focus on SjD as well as the physician’s awareness of muscular involvement.

Consistent with the findings of Colafrancesco et al., EMI typically occurred around the time of SjD onset and was indicated by myalgia as well as bilateral and proximally emphasised muscle weakness (10). Aside from striated skeletal muscles, EMI also involved myocardium, the respiratory system, and the swallowing muscles. It often contributed to severe disease progression, potentially resulting from a reduction of cardiovascular and pulmonary capacity as well as impaired deglutition. Hence, functional signs of muscle weakness need to be evaluated in SjD patients with muscular involvement imperatively (10, 11, 15).

Although muscular pain was detected more frequently in our SjD patients affected by muscular inflammation compared to those without, it needs to be interpreted as a common symptom, appearing in SjD patients without EMI as well (14). Therefore, myalgia should not be considered in isolation, but must be put into context with clinical and diagnostic parameters.

In addition, our findings identified immunological markers, particularly SSA/Ro(52) antibodies and elevated IgG levels, as associated with myositis. Thus, muscular involvement seems to be a condition affecting predominantly seropositive SjD patients, who are often characterised by high antibody production and hypergammaglobulinemia. Nevertheless, further diagnostics including electromyography, magnetic resonance imaging, and muscle biopsy are mandatory to diagnose myositis.

What is more, nerve conduction studies revealed signs of polyneuropathy in 79% of SjD patients with myositis, indicating a possible association of muscular and neural inflammation in SjD. Even though 12/26 SjD patients with myositis were not evaluated electrophysiologically, the prevalence of polyneuropathy within the overall EMI cohort still measured 42%, corroborating a possible link between both disease manifestations. Similar considerations were noted by Konen et al., who detected neuropathic signs in 61% of their patients with idiopathic inflammatory myopathy and SjD (19).

With respect to immunosuppressive therapy, azathioprine, methotrexate, rituximab, and IVIG were commonly used and combined for myositis treatment in our SjD patients, supporting therapeutic approaches, also described by Colafrancesco et al. (10) and Felten et al. (11). Importantly, IgG values were calculated before IVIG treatment to avoid artificially elevated IgG levels resulting from myositis therapy.

According to CK, 76% of our patients diagnosed with SjD and EMI responded to immunosuppressive therapy. However, we reported insufficient therapeutic response in 24% of our SjD patients with myositis. Consequently, muscular inflammation represents a feature of SjD, which appears to be difficult to treat satisfactorily. Nevertheless, the possibility of inadequate patient compliance must be considered. Additionally, the characterisation of SjD patients without response to EMI therapy does not reveal distinct features that allow for clear delineation of this subgroup. Still, there is some evidence suggesting that the therapeutic response is somewhat poorer among male patients than among females (c.f. Figure 4).

Originating from the high frequency of insufficient therapeutic response, there is ongoing research analysing a possible link between SjD and IBM, a myositis subtype that is known to respond insufficiently to immunosuppressive therapy (12, 15, 20–22). Currently discussed hypotheses to explain this association include the presence of antibodies against cytosolic 5′-nucleotidase 1A, analogous patterns of disease pathogenesis (clonal expansion and failure of T-cells), as well as shared key genes (PSMB9, CD74, and HLA-F) and transcription factors (HDGF, WRNIP1) (12, 23, 24).

Another point that should be taken into consideration is the most recent data by Meinecke et al., showing that patients with Ro52 antibody-positive myositis very frequently also present with objectively verifiable sicca symptoms. This suggests that, in such patients, the presence of SjD should be discussed. Consequently, patients with Ro52 antibody-positive myositis should always be evaluated for a possible SjD (25).

### Differences between SjD patients with and without EMI

In contrast to previous findings reported by Felten et al. (11), our SjD patients with EMI did not differ significantly from the average age of 56.0 years at SjD onset. Despite that, we reported a markedly high proportion of male SjD patients, making EMI a manifestation that differs from the disease’s female-dominated sex distribution. Thus, we could not find evidence for EMI, being a disease manifestation that primarily affects women, as suggested by Astouati et al. (15). Similar findings are known for neurological manifestations like polyneuropathy. Seeliger et al. have shown that the sex distribution among these SjD patients was fairly balanced (9, 26).

In addition, our study results suggest that EMI is associated with increased disease activity in SjD, as indicated by elevated constitutional, biological, and total ESSDAI scores, as well as by immunological parameters (e.g., presence of SSA/Ro(52) antibodies, elevated IgG levels) and the frequent use of immunosuppressive agents throughout the course of treatment.

As EMI presents a SjD manifestation with severe impacts and is associated with elevated ESSDAI scores, the high frequency of MClI within the myositis subgroup appears contradictory at first glance. However, these findings may be explained by higher baseline ESSDAI scores, which are typically associated with muscular involvement and are more likely to lead to a decline in disease activity.

Concordant with the findings of Felten et al. (11), the higher levels of disease activity do not contribute to higher ESSPRI scores in follow-up. Potential explanations for this observation include the ESSPRI score’s limited specificity regarding muscular involvement and the high subjectivity inherent in patient-reported evaluations.

Considering the high frequency of concomitant myositis and polyneuropathy, the statistical comparison between SjD patients with EMI and the centre’s overall SjD cohort—whose result may likely reflect a hospital-related bias due to the university’s focus on neurological SjD manifestations—should be interpreted with caution.

The interpretation of inflammatory myopathy in SjD reflects a conceptual divergence between modern myositis classification frameworks and disease-specific activity indices (27, 28). This discrepancy is particularly relevant as overlap syndromes are increasingly recognized within systemic autoimmune diseases.

Recent classification approaches to IIM, aligned with EULAR/ACR principles, categorise myositis occurring in the context of a defined connective tissue disease as overlap myositis. Within this framework, EMI is not considered an isolated entity but rather part of a broader systemic autoimmune overlap. This perspective emphasises shared pathogenic mechanisms and clinical features across connective tissue diseases, supporting a more integrated view of autoimmunity.

In contrast, the ESSDAI adopts a disease-centred approach. In this index, inflammatory myopathy is classified under the “muscular domain” and contributes to the overall activity score of primary SjD. Here, myositis is interpreted as an organ-specific manifestation rather than a separate overlapping condition. This reflects the primary aim of ESSDAI, which is to assess systemic disease activity across organ systems within SjD comprehensively.

The distinction between these frameworks is rooted in their differing objectives. Myositis classification systems aim to define clinically and biologically meaningful subgroups for research and therapeutic stratification, whereas ESSDAI is designed for longitudinal monitoring of disease activity in SjD. As a result, the same clinical manifestation—myositis—can be classified differently depending on context.

In practice, these approaches are complementary rather than conflicting. A patient with primary SjD and inflammatory myopathy may be classified as having overlap myositis while simultaneously being scored as having muscular involvement in ESSDAI. Recognising this dual framework is important for accurate diagnosis, appropriate treatment decisions, and consistent research classification.

Overall, this discrepancy highlights the need for integrative models that reconcile systemic disease classification with organ-based activity assessment, particularly as precision medicine approaches continue to evolve in autoimmune diseases.

### Strengths and limitations

This study is based on a patient cohort, precisely defined and characterised by well-defined organ manifestations and clinical features. We were able to thoroughly delineate EMI in SjD, focusing especially on epidemiology, clinical presentation, diagnostics, and therapy. Thus, our study is among the first ones of its size to focus on EMI in SjD, thereby contributing new clinical data to the field of muscular inflammation. Our findings appear particularly relevant, given the paucity of data on inflammatory myopathy from the rheumatologic perspective of SjD.

In addition, we found that SSA/Ro(52) antibodies and elevated IgG levels were associated with EMI in SjD patients, thereby enhancing immunological diagnostics. These myositis-associated markers may also contribute to a more refined analysis of SjD subgroups, which are gaining relevance in light of ongoing phase III trials, examining the use of immunosuppressive agents and biologicals in SjD. As EMI appears to be a SjD manifestation, predominantly affecting seropositive patients, who are known for more serious disease progression (29, 30), this patient cohort may receive targeted treatment in the future.

Contrary to previous studies on this issue, our SjD EMI cohort is characterised by a higher-than-average proportion of male patients. This can be partially explained by the high proportion of patients with elevated ESSDAI scores, which are often reported with respect to male sex (31). Moreover, our cohort generally includes patients with higher overall and domain-specific ESSDAI scores than typically reported, reflecting the nature of a university hospital setting, where individuals are more frequently referred for complex treatment and specialised medical care.

Notably, a limitation of our study is its retrospective study design. Due to the fact that EMI represents a rare manifestation of SjD, our study is also based on small sample sizes and individualised therapeutic approaches. Due to discrepancies in sex distribution between SjD patients with and without myositis, we opted against increasing the number of matched SjD controls and decided to include a comparison with the centre’s overall SjD cohort to ensure both exact age- and sex-matching and an adequate number of SjD controls. Yet, we cannot rule out a certain bias resulting from the 1:1 matching (28).

Finally, calculating therapeutic response proved challenging. In our patients with SjD and myositis, we gauged response to therapy by CK and MClI. Although both parameters are used, by default, to measure disease progression, they may be influenced by confounding variables and are therefore characterised by a certain degree of unspecificity. Thus, further investigations, preferably prospective studies, are required to assess therapeutic response in patients with SjD and myositis more precisely—for instance, by classifying these patients according to the system of idiopathic inflammatory myopathies and evaluating them by using the total improvement score. Additional research is also needed regarding the association between SjD and IBM, which—owing to its atypical presentation—is frequently difficult to diagnose and was not specifically addressed in our study.

## Conclusion

In summary, our study shows that inflammatory myopathy is a rare but severe manifestation of SjD that typically presents with myalgia, proximally emphasised paresis, and signs of functional muscle weakness. We demonstrated that myositis seems to be a SjD manifestation associated with male sex, SSA/Ro(52) antibody production, hyper-IgG levels, and a high state of disease activity.

Considering that muscular inflammation represents a severe disease condition of SjD, which is often difficult to treat efficiently and characterized in only a few and small studies so far, further research on this topic is required. Our findings reinforce the alignment of SjD-associated myopathy with overlap myositis within modern classification frameworks, while also highlighting a substantial subset of patients with IBM, which may have distinct clinical and prognostic implications.

## Data Availability

The data analyzed in this study is subject to the following licenses/restrictions: The datasets used in this study are not publicly available due to confidentiality and ethical restrictions, but are available from the corresponding author upon reasonable request. Requests to access these datasets should be directed to Ernst.Diana@mh-hannover.de.
